# Protection of Reinforced Concrete Structures of Waste Water Treatment Reservoirs with Stainless Steel Coating Using Arc Thermal Spraying Technique in Acidified Water

**DOI:** 10.3390/ma9090753

**Published:** 2016-09-03

**Authors:** Han-Seung Lee, Jin-Ho Park, Jitendra Kumar Singh, Mohamed A. Ismail

**Affiliations:** 1 Department of Architectural Engineering, Hanyang University, 1271 Sa 3-dong, Sangrok-gu, Ansan 426-791, Korea; ercleehs@hanyang.ac.kr (H.-S.L.); jinho9422@naver.com (J.-H.P.); 2 Department of Chemistry, Indian Institute of Engineering Science and Technology (IIEST), Shibpur, Howrah 711 103, West Bengal, India; 3 Department of Civil and Construction Engineering, Faculty of Engineering and Science, Curtin University Sarawak, CDT 250, Miri 98009, Sarawak, Malaysia; m.abdelkader@curtin.edu.my

**Keywords:** acid, stainless steel, concrete, coating, electrochemical impedance spectroscopy, potentiodynamic

## Abstract

Waste water treatment reservoirs are contaminated with many hazardous chemicals and acids. Reservoirs typically comprise concrete and reinforcement steel bars, and the main elements responsible for their deterioration are hazardous chemicals, acids, and ozone. Currently, a variety of techniques are being used to protect reservoirs from exposure to these elements. The most widely used techniques are stainless steel plating and polymeric coating. In this study, a technique known as arc thermal spraying was used. It is a more convenient and economical method for protecting both concrete and reinforcement steel bar from deterioration in waste water treatment reservoirs. In this study, 316L stainless steel coating was applied to a concrete surface, and different electrochemical experiments were performed to evaluate the performance of coatings in different acidic pH solutions. The coating generated from the arc thermal spraying process significantly protected the concrete surface from corrosion in acidic pH solutions, owing to the formation of a double layer capacitance—a mixture of Cr^3+^ enriched with Cr_2_O_3_ and Cr-hydroxide in inner and Fe^3+^ oxide on the outer layer of the coating. The formation of this passive film is defective owing to the non-homogeneous 316L stainless steel coating surface. In the pH 5 solution, the growth of a passive film is adequate due to the presence of un-dissociated water molecules in the aqueous sulfuric acid solution. The coated surface is sealed with alkyl epoxide, which acts as a barrier against the penetration of acidic solutions. This coating exhibits higher impedance values among the three studied acidic pH solutions.

## 1. Introduction

Waste water contains various types of microbes, including sulfur-reducing bacteria, hazardous materials (i.e., Pb, As, Sn, Cd), and acidic elements, all of which are harmful to living organisms. The waste water can be made potable after treatment. However, owing to the initial presence of these acidic and harmful elements in the wastewater, concrete waste water treatment reservoirs receive direct exposure to them [1]. Concrete is a porous material, which means that aggressive species of waste water are able to diffuse into and out of it, exposing the surrounding ground to contamination [2,3]. Hazardous waste water chemicals affect the quality of concrete and are responsible for its deterioration, as well as the deterioration of the reinforcement steel bars embedded within, after a certain period of time [4]. This deterioration is the result of carbonation, exposure to chloride ions, and acidification. 

Other factors that affect the quality of waste water treatment reservoirs are ozone and activated granular carbon. These chemicals are used for the purification of water in advanced water treatment plants. These chemicals can cause cracking, fading, and spalling of concrete during short periods of exposure [5]. Owing to the cracking and spalling of the reservoir concrete, the reinforcement steel bars start to corrode. Besides ozone and activated carbon, other acidic components are present in waste water. Examples include H_2_S and H_2_CO_3_, which reduce the pH of water from neutral (pH of 7) to acidic (pH of 4). Ozone causes the color of concrete to fade after six years of exposure [5]. Finally, activated carbon is adsorbed on steel surfaces through the pores of the concrete and can cause severe corrosion of the embedded steel bars.

When concrete is subjected to harsh environments for a prolonged period of time, it begins to deteriorate. There are many recommended methods for protecting concrete in waste water reservoirs, including polymeric coatings [6,7] and stainless steel plating [8,9]. The polymeric coating has several drawbacks, including color fading and adhesion due to direct exposure to ozone in waste water treatment reservoir. The thermal contraction and expansion coefficients of polymers and concrete are different, which causes the detachment of the polymeric coating from concrete and can lead to spalling after a period of time. The stainless steel plates are used with grouting and anchoring on the outer surface of concrete. However, this process is not economical, and researchers do not recommend its use. Researchers recommend a stainless steel coating on the outer surface of the concrete of waste water treatment reservoirs. Based on the drawbacks of the protective schemes listed above, it was decided to apply the stainless steel coating using the arc thermal spraying process, as it is convenient, environmentally friendly, economical, and easy to apply.

The arc thermal spray coating can be applied on a clean newly erected or corroded surface. During the arc thermal spraying process, twin wires are used for coating and an electric arc melts the tips of the wires. After arcing, the wires melt and are atomized by hot air, which impinges on the target (substrate) surface. The melted metal droplets adhere onto the substrates and are cooled at room temperature, resulting in the formation of a thick coating [10]. Owing to the high spraying speed and the sudden cooling of the melted metal droplets on the substrate surface, macro pores/defects are formed [11,12,13,14,15] in the coating. 

Waste water treatment reservoirs contain many aggressive species of chemicals and microbes, which leads to the corrosion of reinforced concrete. Therefore, it was decided to protect the reinforced concrete from exposure to these harmful elements using a 316L stainless steel coating applied by arc thermal spraying process. A literature review revealed that research had not yet been performed on the protection of concrete using a 316L stainless steel coating in different acidic pH solutions. 

## 2. Experimental Procedure

### 2.1. Process of Coating

**Figure 1:**
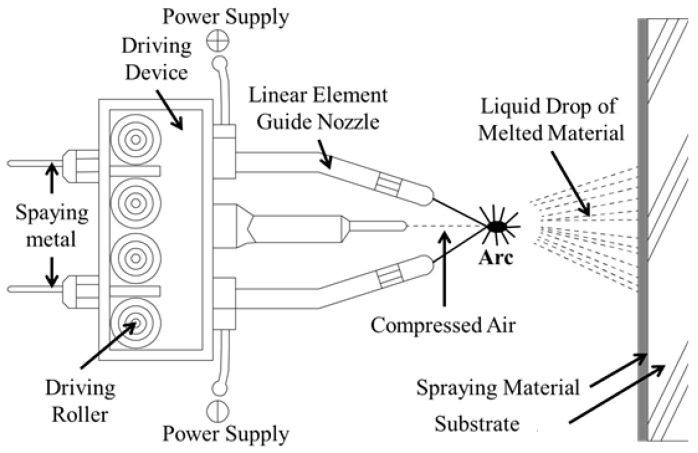
Schematic of the arc thermal spraying process.

The stainless steel coating was applied on the concrete surface using the arc thermal spraying process. The composition of the 1.6 mm wire is shown in Table 1. The wires are mainly comprised of Mn, Cr, Ni, Mo, and Fe, which resembles the composition of 316L stainless steel. The thickness of the coating was measured by an Elcometer 456 dry film thickness gauge (Shinagawa-ku, Tokyo, Japan) at different locations on the concrete—approximately 200 μm (±5 μm). Figure 1 shows that the arc thermal spraying process using the 316L stainless steel coating was applied on the concrete surface with a circular slit of hot and compressed air [16,17,18]. The melted metal diffused and cooled at room temperature and pores were formed in the coating. The spraying of 316L stainless steel on the concrete surface was accomplished by keeping the sample 20 cm away from the spray gun at an air pressure of 6 bars. The spraying voltage and current were maintained at approximately 30 V and 200 mA, respectively [19,20,21,22].

After application of the coating on the concrete surface, the adhesion of the coating was measured according to the ASTM D4541 test method [23]. In this process, a 40 mm × 40 mm section of the coating substrate was taken for an adhesion test. 

### 2.2. Electrochemical Experiments

Electrochemical experiments were carried out on the applied coating and the 316L stainless steel plate. After coating, different processes were employed to smooth the roughened surface of the sprayed coatings. The sprayed coating surface was abraded with 600 μm emery paper to smooth the surface, and alkyl epoxide was used to fill the pores/defects in the sprayed coating. 

**Figure 2:**
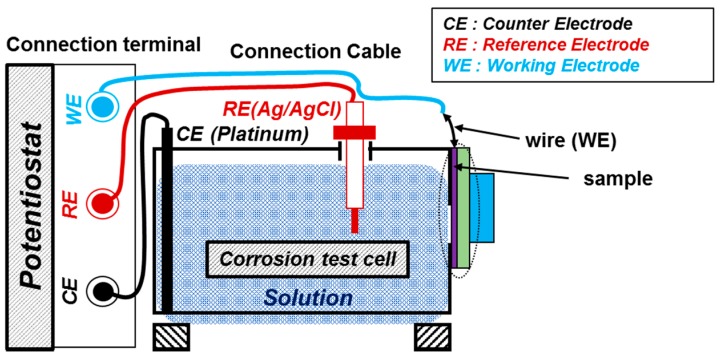
Schematic of the electrochemical setup.

To perform the electrochemical experiments, the solution was prepared in double distilled water by adding a few drops of 0.5 M H_2_SO_4_ to reduce the pH below 7 at 25 °C. Three different pH solutions were chosen for the experiments: pH 6, pH 5, and pH 4. These experiments were performed by three electrode systems (Figure 2), where the coating acted as the working electrode (WE), the platinum wire acted as the counter electrode (CE), and the silver–silver chloride acted as the reference electrode (RE). The WE was soldered with copper wire to the coating. To ascertain a proper electrical connection, a multi-meter was used to measure the resistance because the back side of coating substrate was concrete, which is non-conducting in nature. The area of the working electrode was 0.78 cm^2^, and it was fixed for all the samples. 

The electrochemical impedance spectroscopy (EIS) experiments were carried out by changing the frequency of a 10 mV sinusoidal voltage from 100 kHz to 0.01 Hz. DC polarization experiments were performed from −0.4 V to +0.8 V vs. open circuit potential at 1 mV/s scan rate. The potentiostat was a VersaSTAT (Princeton Applied Research, Oak Ridge, TN, USA), and the data analysis was carried out using the Metrohm Autolab Nova 1.10 software. The 316L stainless steel plate was polished to remove oxides from the surface prior to starting any electrochemical experiments. All electrochemical experiments were carried out in triplicate at room temperature (27 ± 1 °C) to generate reproducible data. 

### 2.3. Characterization of Coating 

The morphology of the coating, plate, and concrete were determined using a scanning electron microscope (SEM, Philips XL 30, North Billerica, MA, USA) operated at 15 kV. Prior to taking the images of coatings using an SEM, they were coated with platinum to increase their conductivity and avoid a charging effect.

## 3. Results

### 3.1. Morphology of Coatings, Plate, and Concrete Surface

**Figure 3:**
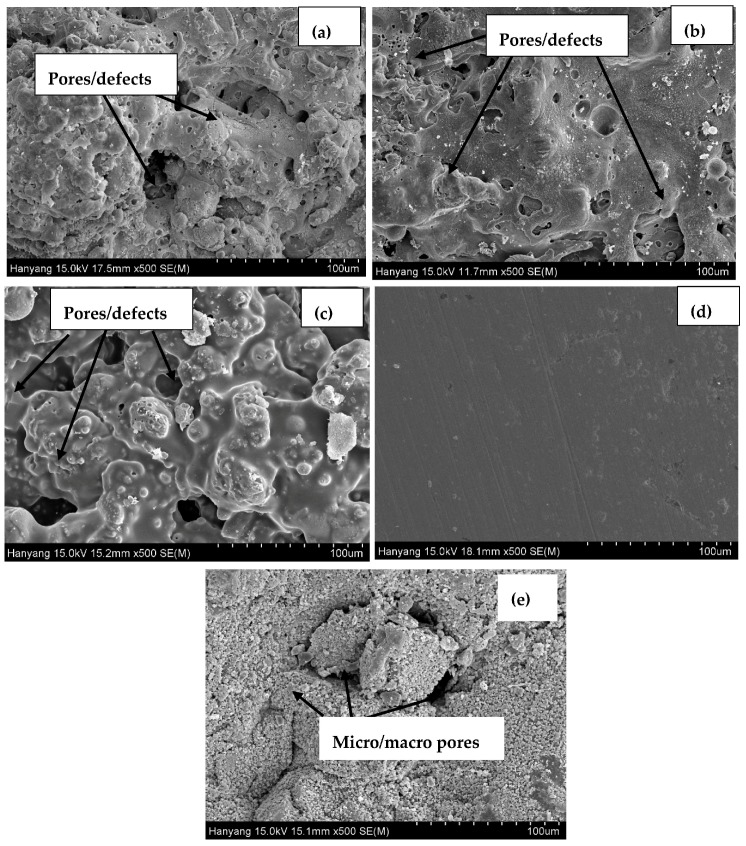
SEM images of 316L stainless steel coating (**a**) sprayed; (**b**) abraded; and (**c**) sealed; (**d**) stainless steel plate; and (**e**) OPC concrete (without coating).

The SEM images of the top surface of the coating, the 316L stainless steel plate, and the concrete surface are shown in Figure 3. The 316L stainless steel sprayed surface is rough, with evidence of defects/pores and unevenness (Figure 3a). This surface facilitates the ingress of aggressive ions, gases, and moisture from the atmosphere, which can later cause deterioration of the coating. To smooth out the sprayed surface, the coatings were polished with emery paper and sprayed with alkyl epoxide to fill the pores/defects. The polished (abraded) and epoxide coated (sealed) surfaces are presented in Figure 3b,c, respectively. The abraded (Figure 3b) surface is slightly smoother than the sprayed surface, owing to the process of polishing with emery paper. The sealed surface (Figure 3c) filled the pores of the coating by alkyl epoxide and worked as a barrier against the penetration of aggressive ions and acidic gases towards the substrate. Figure 3d shows the top surface of the polished 316L stainless steel plate, which is uniform with minimum defects or scratches. The ordinary Portland cement (OPC) concrete morphology exhibited macro- and micro-sized pores connected with circular and elongated holes (Figure 3e). The concrete structure was porous and allowed the ingress of water and other aggressive molecules in and out of the surface. 

The adhesion test on the coating on the concrete surface was measured; the respective standard deviations are presented in Table 2. The adhesion test on the coating was conducted for four samples and the average was calculated. From Table 2, it can be seen that the sprayed and abraded coatings have almost the same average adhesion results, with values of 3.39 MPa and 3.31 MPa, respectively. The adhesion of the sealed surface with the alkyl epoxide coating was 4.06 MPa, which was greater than the sprayed and abraded coatings. This result suggests that the adhesion of alkyl epoxide with a coating substrate was more pronounced. The maximum standard deviation was observed in the sprayed coating substrate (0.21 MPa) while the lowest standard deviation was observed in the sealed surface (0.06 MPa). The pull-off adhesion tester was used on the coating surface by increasing the load on it; the adhesion was measured at certain applied loads when the coating detached from the surface. The adhesion of the coating was lower than what is recommended in ASTM D4541 [23], which can be attributed to the selection of a larger coating surface area (16 cm^2^). 

### 3.2. Electrochemical Experiments for Different pH Solutions

#### 3.2.1. EIS Experiments for Different pH Solutions

**Figure 4:**
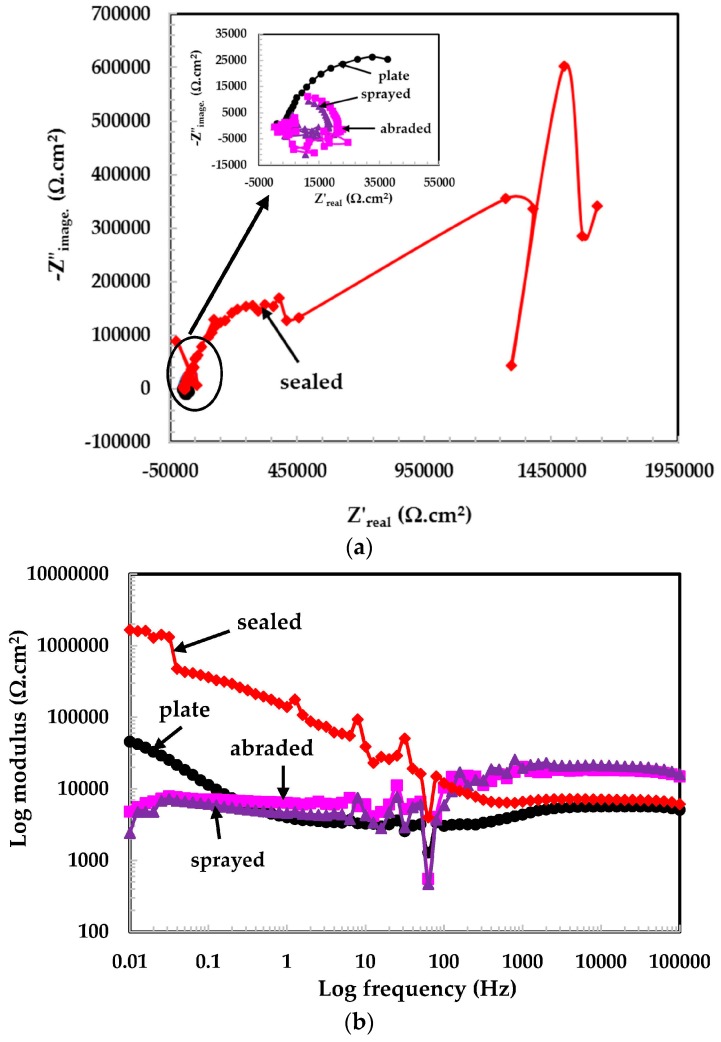

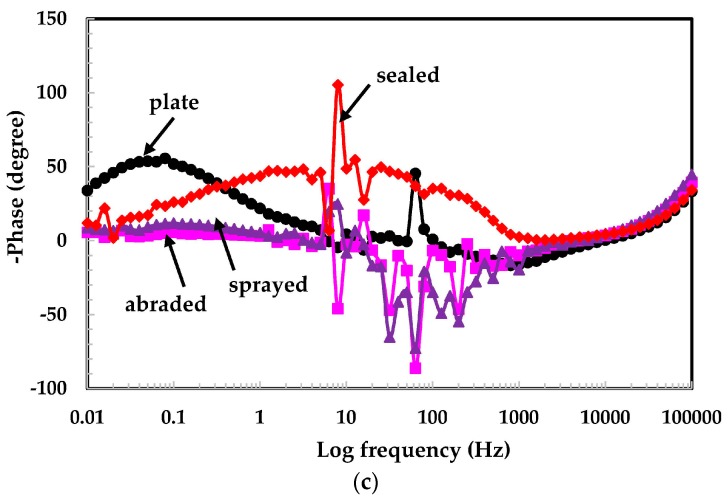
(**a**) Nyquist plots of 316L stainless steel coating applied by a spraying in pH = 6; (**b**) log modulus-frequency Bode plots of the 316L stainless steel coating applied by arc thermal spraying in pH = 6; (**c**) phase-frequency Bode plots of 316L stainless steel coating applied by the arc thermal spraying process at pH = 6.

**Figure 5:**
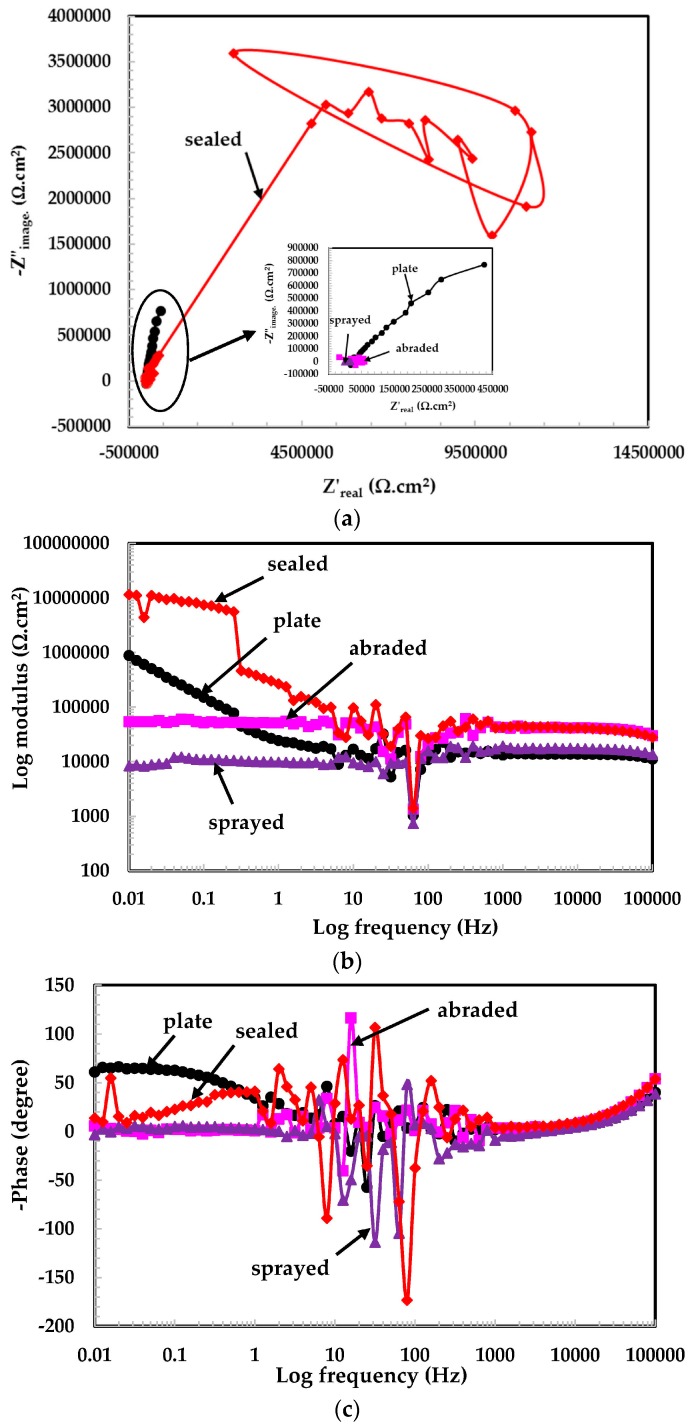
(**a**) Nyquist plots of 316L stainless steel coating applied by arc thermal spray process at pH = 5; (**b**) log modulus–frequency Bode plots of 316L stainless steel coating applied by arc thermal spray process at pH = 5; (**c**) phase–frequency Bode plots of 316L stainless steel coating applied by arc thermal spray process at pH = 5.

**Figure 6:**
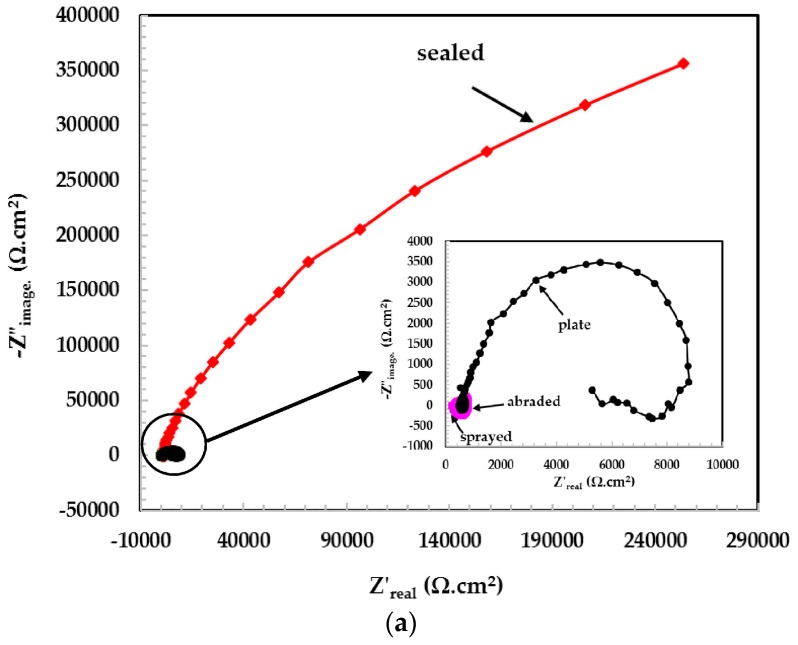

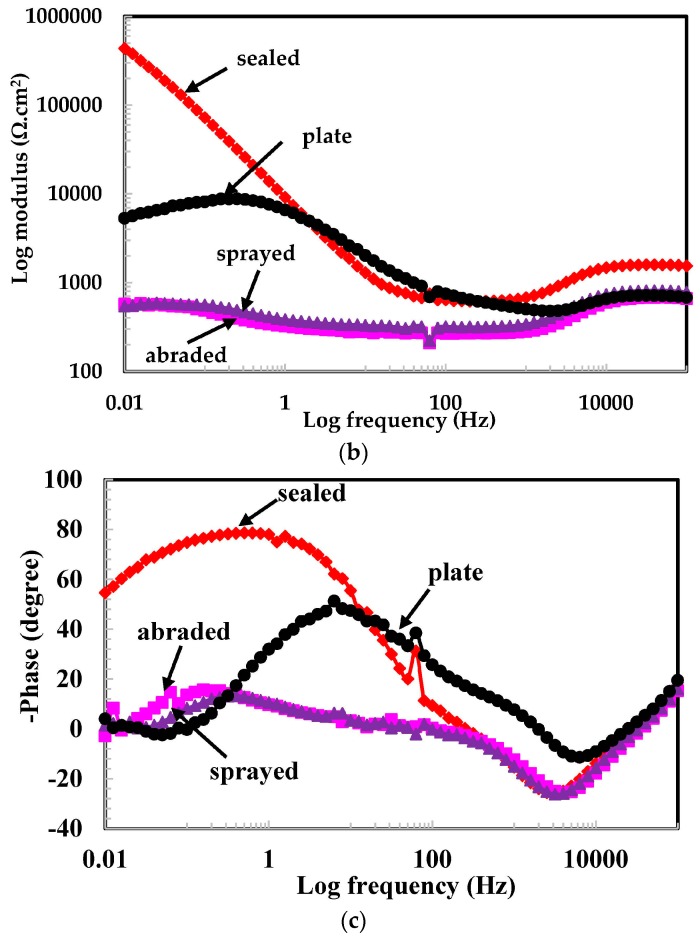
(**a**) Nyquist plots of 316L stainless steel coating applied by arc thermal spraying at pH = 4; (**b**) log modulus–frequency Bode plots of 316L stainless steel coating applied by arc thermal spraying at pH = 4; (**c**) phase–frequency Bode plots of 316L stainless steel coating applied by arc thermal spraying at pH = 4.

The EIS study is an established and powerful technique used to study the phenomena occurring at the metal/solution interface at open circuit potential [24]. The EIS experiments on the top surface of the coatings, and the 316L stainless steel plate were carried out in solutions of different pH values and are shown in Figure 4, Figure 5 and Figure 6. In most of the EIS plots, it can be seen that the graphs are scattered owing to the low conductivity of the solutions and the capacitive properties of passive layers in intermediate frequency ranges. These passive layers contain double layers, a mixed Cr^3+^ enriched with Cr_2_O_3_ layer, Cr-hydroxide in the inner layer, and Fe^3+^ oxide in the outer layer, [25]. The scattered graph in the intermediate frequency range is due to the defective passive film and the high capacitive response. This type of plot was also observed by other researchers [24,25,26,27]. 

The Nyquist plots for the 316L stainless steel coating and plate tested in the pH 6 solution are shown in Figure 4a. A typical Nyquist plot is observed for the sealed and plate samples. As the dimensions of a Nyquist plot are increased, the resistive properties of the samples also increase. This observation is noted in the sealed surface of the coating in the pH 6 solution. The Nyquist plots for the sprayed, abraded, and plate surface samples can be shown in the inset of Figure 4a because their dimensions are very small and are suppressed owing to the sealed coating plot. The scattered data results are observed in this pH solution owing to the reduced conductivity of the solution and the capacitive properties of passive films. The tendency of formation of the passive film is the same for all coatings and is seen in the Nyquist plots.

The EIS graphs are plotted from 100 kHz to 0.01 Hz and the total impedance values are measured at the lowest studied frequency (i.e., 0.01 Hz). Figure 4b shows the log modulus—frequency Bode plots of coatings exposed in the pH 6 solution. From Figure 4b, it is observed that the sealed surface of the coating exhibited the highest impedance values of all the coatings because it works as a barrier against the penetration of the solution. However, the low impedance for the 316L stainless steel plate is due to the oxide layer that is removed because of the polishing surface. Therefore, the surface became bare and began to deteriorate when it came into contact with the solution. The solution resistance (*R_s_*) at the highest frequency considered (100 kHz) is too high because the conductivity of distilled water is lower at 25 °C, which causes additional resistance to electrochemical reaction. There is no considerable difference observed in the impedance values of the sprayed and abraded surface of coatings and they are almost the same as *R_s_*. This may be due to the very low reactivity of the solution with the coatings.

The phase-frequency Bode plots in the pH 6 solution are shown in Figure 4c. The sealed and plate surfaces exhibiting two maxima in different frequency ranges indicate the formation of a capacitance on the corroding interface in middle frequency and passive film formation at lower studied frequency, while the abraded and sprayed surfaces show only one-time constant attributed deterioration property of coating at lower frequency with low angle shift. Figure 4c shows the resistive properties in high frequency, the capacitance at middle frequency, and the broad peak in the low frequency region, which indicate the formation of a passive film [28]. The plate surface sample maxima are shifting about −50° at a lower frequency, which is attributed to the formation and growth of the passive film [29]. 

The Nyquist plots for the samples exposed in the pH 5 solution are shown in Figure 5a. The dimensions of the Nyquist plots for the sealed samples are the highest, which is attributed to the formation of a highly stable passive film and un-dissociated water molecules in the pH 5 solution. At the low frequency the plots are scattered, which may be due to the high resistance of the passive film. This passive film might be non-conducting in nature and cause more resistance to the deterioration of the surface. Another reason may be the non-conducting nature of alkyl epoxide. The Nyquist plots for the other samples are shown in the inset of Figure 5a. The 316L stainless steel plate surface exhibited a larger semi-circle loop than the abraded and sprayed surfaces, which is attributed to the fact that they are more resistant to corrosion in a pH 5 solution than abraded and sprayed samples. Other coating surfaces, i.e., abraded and sprayed samples that exhibit more defects, are susceptible to corrosion in a solution of pH 5. 

The log modulus–frequency Bode plots in the pH 5 solution are shown in Figure 5b. It was observed that the total impedance values for all coatings are very high and pronounced to provide a higher degree of protection. It is possible that in this particular pH solution, the un-dissociated water molecules exist in a high concentration and thus passivate the surface significantly. Other researchers had also discovered that the solution containing aqueous H_2_SO_4_ contains un-dissociated water molecules that passivate the stainless steel surface significantly [30]. From the log modulus–frequency plots, (Figure 5b) it can be seen that the sealed surface exhibits a higher impedance value than the plate, followed by the abraded and sprayed coating.

The phase–frequency Bode plots (Figure 5c) show the higher capacitive property of the film that formed on the corroding interface [31]. The sealed coating and 316L stainless steel plate show broad peaks from the mid to the lower frequency range, which is attributed to passivation and response to capacitive properties. The passive film formed in this pH solution is compact, adherent, and non-hydrated [32]. It may be that more Fe^3+^ and Cr^3+^ ions exist in this passive film. The presence of these ions in the film is attributed to its enhanced resistance to corrosion.

The Nyquist plots of the samples in the pH 4 solution are shown in Figure 6a. It can be seen that the dimensions of the semi-circular loop for all the studied samples are smaller than in the other pH solutions. It is possible that in such acidic pH solutions, the coating started to deteriorate, resulting in a smaller semi-circular loop. This pH solution is, therefore, more susceptible to corrosion, as all the samples exhibited deterioration. There is an inductive loop found in the plate sample due to the diffusion of corrosive products from the surface.

As the pH decreases, the total impedance values are decreased owing to the reduction in pH of the solution. The synergistic effect was caused by H^+^ and SO_4_^2−^ ions, which enhances the corrosion of the corroding interface. This type of observation is found from Figure 6b in the lowest studied pH solution. It is obvious that the deterioration/corrosion of a metallic surface increases at fixed instances and depends on the material, pH of the solution, solution content, and temperature [29,33]. A considerable decrease in impedance value is observed from Figure 6b, and it is the reduction in the pH of the solution that causes the deterioration of the coatings. The results show that the sealed surface performed better than the plate, followed by the abraded and sprayed surfaces.

The phase angle shifted towards a lower frequency with a decrease in angle. This may be attributed to the increase in the anodic surface area of the corroding interface and a decrease in the impedance value (Figure 6c). It is observed that all coatings in the pH 4 solution exhibit two maxima at the lower and higher frequencies. At lower frequency, this phenomenon is attributed to corrosion. At higher frequency, corrosion products blocked the pores/defects of the coating and filled the pits [34]. The phase angle for the sealed surface is shifted towards −75° to −80° (Figure 6c) at lower frequency, and it is confirmed that these surfaces have less anodic surface area than the plate surface, followed by the abraded and sprayed surfaces. The lesser anodic surface area for the sealed surface may be due to alkyl epoxide working as barrier against the penetration of the solution into the coating substrate. The phase angle shifted towards higher frequency for the sprayed and abraded samples due to the deposition of corrosion products on the coating surface at approximately 20° to 25°. The 316L stainless steel plate surface exhibiting approximately 12° at lower frequency might be due to initial corrosion in such a low pH solution. It can be seen from Figure 3d that there are fewer defects/scratches present on the plate surface than on other coatings. Therefore, the corrosion products’ deposition was less on 316L stainless steel plate surface than on abraded and sprayed surfaces. 

**Figure 7:**
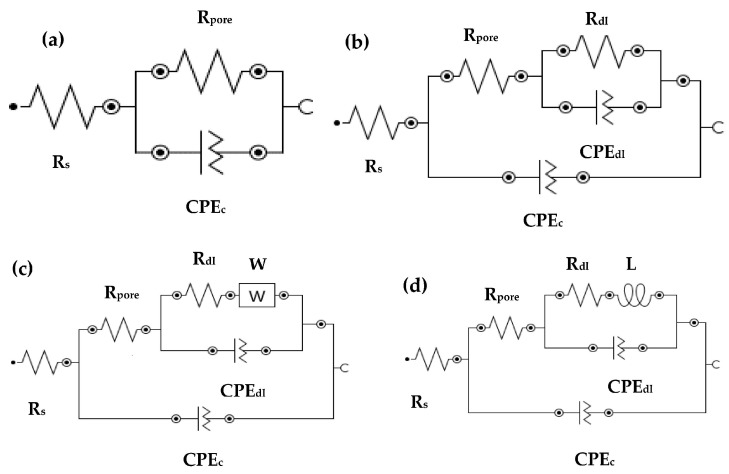
EEC for different coatings exposed in different pH solutions: (**a**) pH 6 for sprayed, abraded, sealed, and plate; as well as pH 5 and 4 for sprayed and abraded; (**b**) pH 5 and 4 for sealed; (**c**) pH 5 for plate; and (**d**) pH 4 for plate.

The electrical equivalent circuit (EEC) for tested samples in different pH solutions is presented in Figure 7. Figure 7a shows the EEC for sprayed, abraded, sealed, and plate surfaces at pH 6, as well as at pH 5 and pH 4 for sprayed and abraded samples. The polarization resistance (*R_pore_*) and constant phase element for coating (*CPE_c_*) are parallel to each other. The sealed samples exposed in pH 5 and pH 4 solutions and their EEC are shown in Figure 7b. The sealed surface coating exposed to pH 4 and pH 5 exhibited another constant phase element (*CPE_dl_*) and resistance (*R_dl_*) for double layer due to the formation of the passive film. The *R_dl_* and *CPE_dl_* in parallel are associated with *R_pore_* in series. Warburg impedance (W) is observed in a pH 5 solution for plate samples owing to the diffusion of ions from the surface and caused resistance (Figure 7c). The inductive loop is observed in the Nyquist as well as in the phase–frequency Bode plot of the 316L stainless steel plate sample exposed to a pH 4 solution (Figure 7d). 

The electrochemical parameters of the samples are extracted from impedance plots in different pH solutions after fitting them in suitable EEC. This is shown in Table 3. In a pH 6 solution, the *R_s_* is almost the same as *R_pore_* owing to the low conductivity of the solution for sprayed and abraded samples. This pH solution is mildly acidic and did not affect the corrosion resistance properties of samples. The *R_pore_* for the sealed sample is much higher than for the plate and other samples. The lower *R_pore_* for the sprayed and abraded samples is due to the formation of more defects on the coating surface during the process of deposition, which facilitates the ingress of acidic ions. The samples tested in pH 5 exhibit the highest *R_pore_* and *R_dl_* values for sealed surface. From Table 3, it is observed that the *R_s_* value for the samples exposed to the pH 6 and 5 solutions is the highest, decreased in pH 4 owing to the highly acidic nature of solution. In the pH 4 solution, more ions are involved, which increases the conductivity of the solution. Therefore, the *R_s_* is lower in a pH 4 solution. The samples exposed to the pH 5 solution exhibit the highest *R_s_* and *R_por_*_e_ values among the other studied pH solutions. The higher *R_s_* and *R_pore_* of the samples in the pH 5 solution might be owing to un-dissociated water molecules and the formation of a highly resistant passive film, respectively. It can be seen from Table 3 that the admittance (*Y_o1_* and *Y_o2_*) for the sealed and plate samples have minimum values, and the dispersion coefficient (α_1_ and α_2_) is more evident in a pH 5 solution. The larger α (around 0.90) indicates that the surface has become more homogenized with a minimum amount of defects in the passive film. The presence of W in the 316L stainless steel plate exposed to a pH 5 solution indicates that the Cr^3+^- and Fe^3+^-enriched ions diffuse from the inner and outer surfaces of the passive film. The sample exposed to the pH 4 solution exhibited a lower *R_por_*_e_ for the sprayed and abraded samples owing to the more acidic nature of the solution and the presence of more defects/pores on the top surface of the coating, which induces deterioration. The presence of inductance (L) for the plate sample exposed to a pH 4 solution is due to the partial breakdown of the passive film and an initial deterioration process.

#### 3.2.2. Potentiodynamic Experiments for Different pH Solutions

**Figure 8:**
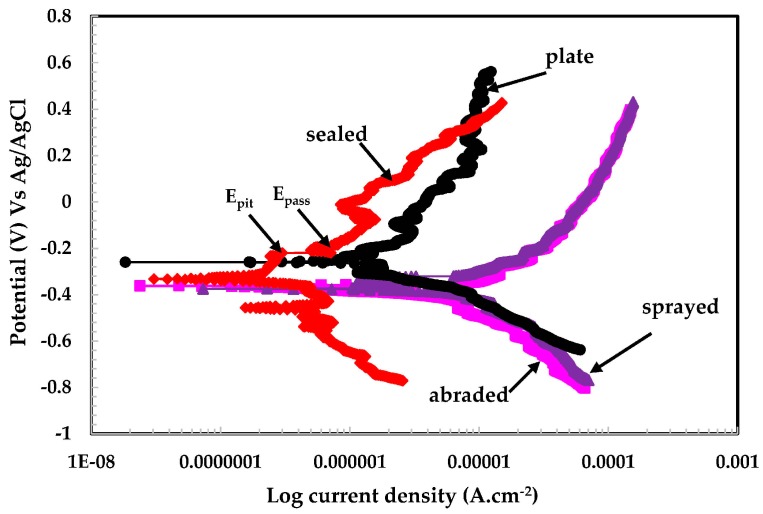
Potentiodynamic plots of 316L stainless steel coating applied by arc thermal spraying at pH = 6.

**Figure 9:**
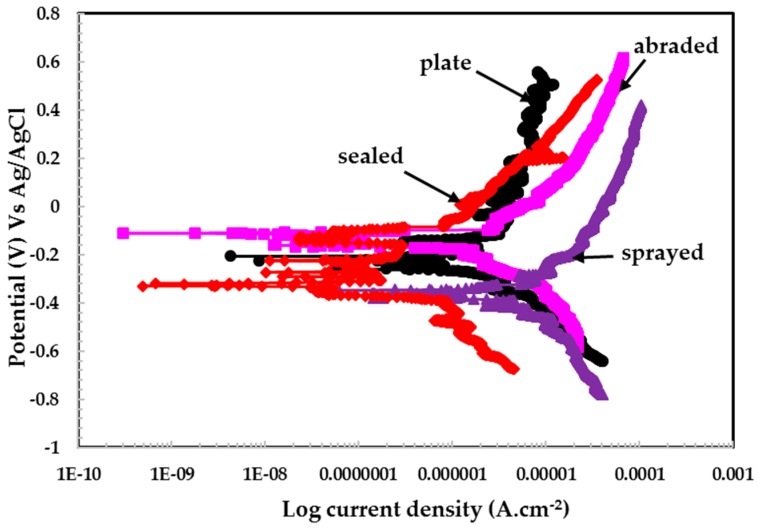
Potentiodynamic plots of 316L stainless steel coating applied by arc thermal spraying at pH = 5.

**Figure 10:**
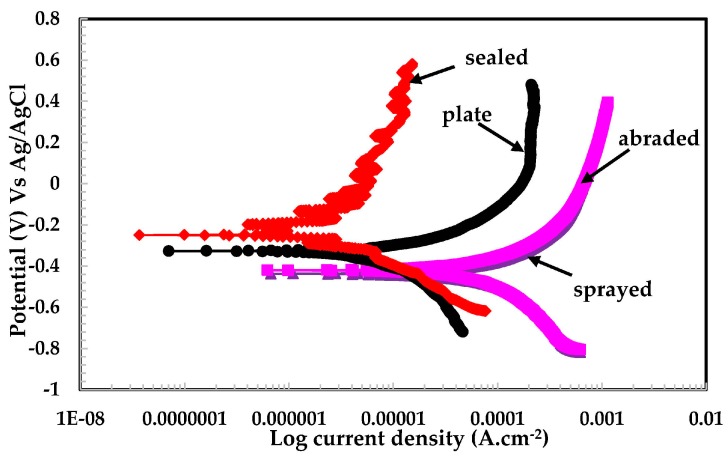
Potentiodynamic plots of 316L stainless steel coating applied by arc thermal spraying at pH = 4.

The potentiodynamic experiments on coatings and 316L stainless steel plate in different pH solutions are shown in Figure 8, Figure 9 and Figure 10. The potentiodynamic experiments on coatings exposed to pH 6 are shown in Figure 8. The sealed sample exhibits a lower current density than the plate, abraded, and sprayed surfaces. The corrosion potential (*E_corr_*) for the sealed surface shows higher activity than the stainless steel plate. This may be attributed to the reaction of the acidic solution with alkyl epoxide, which activated the surface. 

The sealed surface showed passive potential (*E_pass_*) and passive pitting potential (*E_pit_*), along with a decrease in current density, while other coatings did not show this type of behavior in the pH 6 solution. It is observed that the plate surface exhibited higher anodic current than the sealed surface. The sprayed and abraded surfaces show a passive region, but the current density is higher than plate and sealed surfaces. The higher current density for the sprayed and abraded surfaces is due to the presence of more active sites (i.e., pores/defects) on the surface, which enhances the chemical reaction. 

The plate, sprayed, and abraded surfaces show identical reduction properties for passive film during cathodic scanning, which indicates that the formation of the passive film exhibits the same characteristics in a pH 6 solution. Therefore, the sprayed and abraded surfaces showing more anodic current density than the plate and sealed coatings can be attributed to reduced oxide films. However, the plate surface does not have any defects/pores on its surface. Therefore, it indicates that the plate surface contains more enhanced corrosion resistance than the sprayed and abraded surfaces. The scattered curve obtained for the pH 6 solution may be due to the breaking and re-passivating of the film, especially in the case of the plate surface; on the sealed surface it is due to the presence micro-pores in the alkyl epoxide coating. This phenomenon occurs through all scanning ranges of potentiodynamic experiments. 

From Figure 9, it can be seen that the samples exposed to the pH 5 solution exhibited a high degree of protection. The sealed and plate surfaces show metastable potential, which may be due to the fact that the passive film was forming and breaking simultaneously during the scanning process. The sealed surface might contain very fine porous structures, by which the solution can penetrate below the alkyl epoxide to where stainless steel coating was applied. 

This stainless steel coating reacts with aqueous H_2_SO_4_ in a pH 5 solution and forms a passive layer. This layer is resistant to further attack from any aggressive ions on the surface. It is also observed from EIS plots (Figure 5) and Table 3 that sealed surfaces showed higher impedance than others because alkyl epoxide works as a barrier against the penetration of the solution. The stainless steel plate exhibited a greater pitting tendency than the other samples studied, which might be due to the presence of a defective passive film formed while breaking down simultaneously, forming a passive layer. This is a continuous process, therefore it has showed breakdown potential at different scanning ranges. 

The sealed surface shows a greater breakdown potential in the pH 4 solution than the other surfaces (Figure 10). The alkyl epoxide may be susceptible to an acid solution and cause dissolution resultant formation of meta-stable potentials. The sealed surface may be dissolved, or more defects/pits may form as a result of the chemical reaction of epoxide with the acidic solution. 

The plate, abraded, and sprayed surfaces showed the formation of a passive film and corrosion products, deposited in the defects/pits of the coating surface. However, corrosion product enhances the passivation in a pH 4 solution. The sealed surface shows nobler positive *E_corr_* and less corrosion current density (*I_corr_*) than other samples, which attributed high degree of protection. 

An interesting observation was noted while analyzing the results of this experiment. After conducting potentiodynamic experiments on the sprayed and abraded samples, the solutions changed from having no color to yellow or green. This may be due to an increased dissolution rate of the stainless steel coating, resulting in the formation of iron hydroxide in a pH 4 solution. This indicates that in this solution, the corrosion products again were deposited on the coating surface and formed a passive layer [35].

## 4. Discussion

Sulfur-reducing bacteria are present in waste water (sewer water), resulting in the reduction of SO_4_^2−^ into sulfur. Owing to the presence of sulfur, water can become acidic. To simulate this condition, H_2_SO_4_ was used in this study.

The conductivity of the solution (pH 6) is low, but can be increased by adding salt. There was no salt or ion present beforehand, except for a few drops of 0.5 M sulfuric acid in all of the solutions focused on in this study. The pH was reduced by adding a few drops of 0.5 M H_2_SO_4_, which is incapable of increasing the conductivity of the solution. The conductivity of distilled water is low—approximately 10 × 10^−6^ W^−1^ m^−1^ (20 dS/m). In all the other instances in this study, it was observed that the solution’s resistance is high. In the presence of H_2_SO_4_, stainless steel forms a protective passive film that behaves like a semiconductor.

Banas and Stypula have observed that in aqueous H_2_SO_4_ acid solutions, austenitic steel is protected by the formation of an oxyhydroxide and oxide–hydroxide passive film due to un-dissociated water molecules in solution [30]. Lai et al. studied the characteristics of the formation of passive films on AISI 304 stainless steel in a 0.5 M H_2_SO_4_ solution and found that steel is composed of oxyhydroxides, Fe_2_O_3_, FeO, Cr_2_O_3_, NiO, sulfate, sulfite, and sulfide (FeS, NiS) [36]. The stability of passive films on stainless steel can vary, depending on the composition of alloys, environment, film thickness, and structure [37]. This passive film is partially hydrated; this was observed by Asami et al. [38].

There is a general agreement in the literature that the passive film of stainless steel in aqueous H_2_SO_4_ is due to the formation of Cr^3+^ compounds on the upper surface of the steel. This layer is of nanometer thickness and is enriched with chromium [39]. The thickness and composition of passive films can be altered by changing the pH and chloride content of the solution, and the composition of the steel. Elsener and Rossi have stated that when the pH of a solution is changed, it changes the tendency of the formation of passive films. As the pH was increased, the passive film became hydrated and more protective, as shown in this study [40].

**Figure 11:**
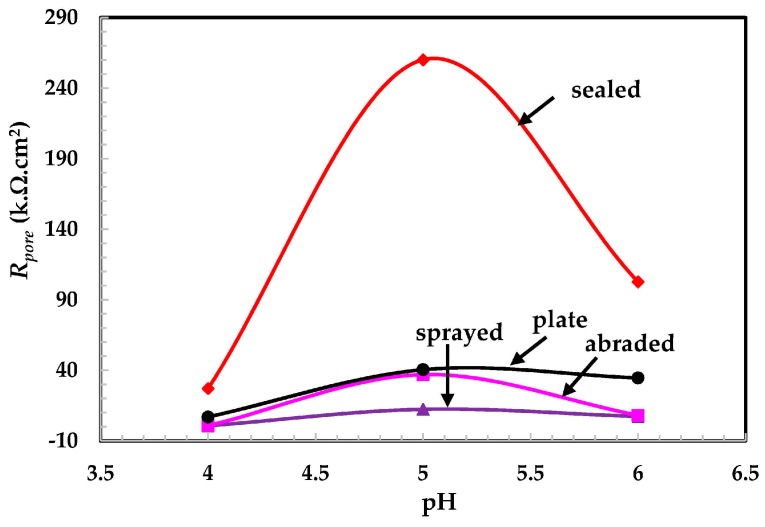
pH and *R_pore_* plots of coatings exposed to different pH solutions.

The electrochemical parameters are extracted from potentiodynamic plots in different pH solutions by fitting the graph in a Tafel slope. The *R_pore_* and other parameters are presented in Figure 11 and Table 4. In the pH 5 solution, the polarization resistance of the passive film (Figure 11) for all studied samples was larger than in other pH solutions. This may be due to an increase in the film thickness, increased oxidation of iron, reduced Cr^3+^ [41], and un-dissociated water molecules [30]. Alhosseini and Vafaeian have reported that passive films in sulfuric acid behave as semiconductors and they found that AISI 304 and AISI 430 steel formed n- and p-type semiconductors, respectively [42]. As the pH is reduced, the *R_pore_* decreased dramatically. It is possible that the synergistic effect caused by the pH 4 solution resulted in greater corrosion of the coating surface. 

It can be seen from Figure 11 that at a pH of 4, the *R_pore_* values of the plate, abraded, and sprayed samples are almost the same. This indicates that the coating plays a significant role in protecting concrete against deterioration. This coating is more economical, while the stainless steel plate is very costly. In pH 5, the *R_pore_* for abraded and plate surfaces are the same. This result indicates that this coating provides protection up to a certain extent of the acidic pH solution and works as effectively as the stainless steel plate coating. After the abrading of the sprayed surface, the pores/defects of the sprayed coating surface get filled, which reduces the active surface area and results in the formation of a more compact and uniform coating layer (Figure 3). Therefore, the abraded surface had a higher *R_pore_* value than the sprayed coating surface. The highest *R_pore_* was observed in the sealed surface for all studied pH solutions owing to the alkyl epoxide working as a barrier to the penetration of the acidic solution into the substrate. 

Owing to the presence of more active sites on the sprayed sample, it exhibited more active (negative) potential for all of the tested pH solutions than the other samples (Table 4). It was observed that all of the coatings showed active potential in the pH 4 solution, which may be due to the more acidic nature of the solution. The dissolution of the surface is enhanced in the pH 4 solution, while at pH 6 it is decreased. 

An interesting result was observed in the case of the pH 5 solution: *E_corr_* values for all samples are nobler than other pH solutions. It might be that, in this solution, the surface became passive and resisted the solution’s penetration into the coating surface. This result corroborates the finding of a corrosion current density (*I_corr_*) of coatings in the pH 5 solution. The lowest *I_corr_* value is observed at pH 5 for all studied coatings and may be due to the formation of a passive film that protects the surface. The corrosion rate (μm·year^−1^) was calculated using the following equation [43]:

(1) $$\mathrm{Corrosion}\;\mathrm{rate}\;\left(µm/y\right)\;=\frac{\;3.27\;\;\times \;\;I_{corr}\;\times \;\;E.W.}{d}.$$

The corrosion rate in Equation (1) is expressed in micrometers per year (μm/y); *I_corr_* is in μA/cm^2^. The *I_corr_* was obtained by dividing the total surface area of the working electrode by the corrosion current. *E.W.* represents equivalent weight (g/mole) and d is density (g/cm^3^).

The corrosion rate was the highest in the pH 4 solution, which may have been caused by the aggressiveness of the solution. The sprayed coating showed the highest corrosion rate due to the dissolution of the coating and the penetration of the solution through the pores of the coating (Table 4). Therefore, the highest corrosion rate is observed in the pH 4 solution. It is observed from the EIS and potentiodynamic plots that the coatings showed the highest corrosion resistance in the pH 5 solution. The EIS results agree with electrochemical parameters extracted from potentiodynamic plots (Table 4). The error observed in *E_corr_* and *I_corr_* during the fitting of the potentiodynamic plots in the Tafel region is presented in Table 4 in brackets. A 5% maximum error was observed during the fitting of the potentiodynamic curve for *E_corr_* and *I_corr_*. 

**Figure 12:**
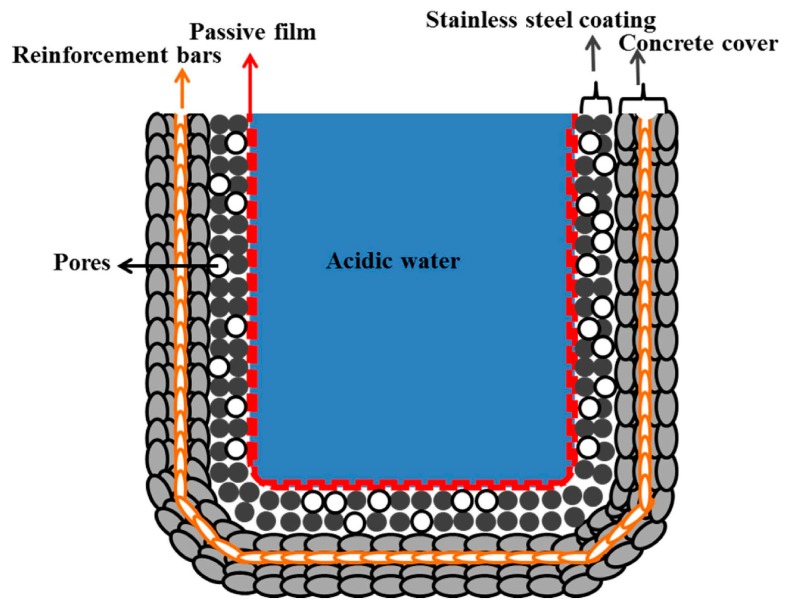
Schematic of the formation of a passive film on the stainless steel coated surface of a water treatment reservoir. The coating was applied by arc thermal spraying and the water is acidic in nature.

The protective mechanism of stainless steel coatings is described by the schematic presented in Figure 12. The outer surface of a water treatment reservoir is made of concrete embedded with reinforcement steel bars, which provides added strength to the overall structure. 

Waste/acidic water is stored in concrete reservoirs for purification and this contaminated water is purified by different processes. Therefore, the waste water affects the health of the concrete at longer durations of treatment. To protect the concrete from hazardous materials in waste water, a stainless steel coating was applied on the inner surface of the reservoir using the arc thermal spraying technique. This coating is porous in nature and, as a result, different techniques have been used to minimize the porosity of the coating. 

Owing to the presence of hazardous chemicals in waste water, the pH of the water can reach acidic levels, comprising mostly sulfuric acids. To simulate this acidic environment, distilled water was acidified using 0.5 M H_2_SO_4_ acid. The stainless steel coating on the inner side of the reservoir reacted with the acidified water solution and formed a passive film, which provides further protection from corrosion. If a metallic coating other than stainless steel were used, it would be vulnerable to the acid solution and thus dissolve. This passive film can exist up to a mild acidic medium and below the critical pH level. The highly acidic pH solution destroys the passive film and causes deterioration. 

## 5. Conclusions 

Based on the above results and discussion, the following conclusions can be drawn:

1. The 316L stainless steel coating was applied to the concrete surface of a waste water reservoir, which provides protection across a variety of acidic pH solutions.
2. The alkyl epoxide sealed coating provided the best protection against contamination because it worked as a barrier against the penetration of acidic solutions.
3. The scattered impedance plots in the acidic pH solutions tested were due to the formation of capacitance and defective passive films at intermediate frequencies.
4. The different values of pH of the aqueous H_2_SO_4_ acid solution passivated the 316L stainless steel coating because of the formation of Cr-enriched phases on the top surface.
5. The lowest polarization resistance of the coatings in pH 4 was because of the aggressiveness of the solution, which leads to the dissolution of the coating.
6. Defects in the surface of the coating made the coating susceptible to corrosion.
7. The presence of macro cells in the sprayed and abraded coatings resulted in the formation of pits and galvanic cells, which caused the coating to corrode in the acidic solution.
8. In the pH 5 solution, the abraded coating exhibited almost the same *R_pore_* value as the 316L stainless steel plate coating owing to the formation of a stable passive film. This passive film is formed in the presence of un-dissociated water molecules of the aqueous H_2_SO_4_ solution.

## Figures and Tables

**Table 1:** Composition of 316L stainless steel wires used in coating.

Elements (wt %)									
C	Mn	Si	Cr	Ni	Mo	P	S	N	Fe
0.03	1.940	0.700	16.790	9.600	1.800	0.040	0.025	0.010	balance

**Table 2:** Bond adhesion test results of the 316L stainless steel coating on the concrete surface applied using the arc thermal spraying process.

Sample Details	Bond Strength (MPa)				Average (MPa)	Standard Deviation (MPa)
	1	2	3	4		
Sprayed	3.57	3.56	3.29	3.14	3.39	0.21
Abraded	3.33	3.22	3.19	3.49	3.31	0.14
Sealed	4.12	4.09	4.02	3.99	4.06	0.06

**Table 3:** Electrochemical parameters of coatings and plate in different acidic pH solutions are extracted after fitting of electrochemical impedance spectra in suitable EEC.

pH	Sample Details	*R*_S_	Electrochemical Parameters						W	L
			CPE_c_			CPE_dl_				
		kΩ·cm^2^	*R_pore_* kΩ·cm^2^	*Y_o_*_1_ (1 × 10^−4^) (Ω·cm^−2^·s^−n^)	*α*_1_	*R_dl_* kΩ·cm^2^	*Y_o_*_2_ (1 × 10^−6^) (Ω·cm^2^·s^−0.5^)	*α*_2_	(1 × 10^−4^)	(H·cm^2^)
6	Sprayed	1.32	1.47	7.94	0.74	-	-	-	-	-
	Abraded	1.27	2.01	7.80	0.79	-	-	-	-	-
	Sealed	1.09	607.00	0.01	0.94	-	-	-	-	-
	Plate	1.02	69.42	1.45	0.83	-	-	-	-	-
5	Sprayed	1.37	1.97	1.54	0.95	-	-	-	-	-
	Abraded	2.18	4.37	0.01	0.95	-	-	-	-	-
	Sealed	1.94	787.00	0.01	0.99	356.00	0.90	0.90	-	-
	Plate	1.25	570.00	0.11	0.98	110.00	1.32	0.89	1.10	-
4	Sprayed	0.35	0.37	22.69	0.70	-	-	-	-	-
	Abraded	0.30	0.35	28.32	0.70	-	-	-	-	-
	Sealed	0.55	563.00	0.21	0.90	173.00	1.10	0.89	-	-
	Plate	0.32	103.00	0.21	0.89	88.00	1.19	0.88	-	25.63

**Table 4:** Electrochemical parameters of coatings and plate are extracted from the Tafel region of potentiodynamic plots in different acidic pH solutions.

pH	Sample Details	Electrochemical Parameters		Corrosion Rate (μm·year^−1^)
		*E_corr_* (V) Vs Ag/AgCl	*I_corr_* (μA·cm^−2^)	
		(Error, V)	(Error, μA·cm^−2^)	
6	Sprayed	−0.380 (0.011)	17.56 (0.60)	200.43
	Abraded	−0.366 (0.020)	8.52 (0.40)	97.24
	Sealed	−0.336 (0.013)	0.32 (0.01)	3.65
	Plate	−0.244 (0.012)	1.56 (0.07)	17.80
5	Sprayed	−0.369 (0.019)	8.27 (0.10)	94.39
	Abraded	−0.156 (0.010)	1.33 (0.03)	15.18
	Sealed	−0.278 (0.011)	0.11 (0.006)	1.25
	Plate	−0.219 (0.011)	0.74 (0.02)	8.44
4	Sprayed	−0.444 (0.020)	1124.5 (20.50)	12,834.76
	Abraded	−0.413 (0.020)	504.83 (8.09)	5762.00
	Sealed	−0.255 (0.015)	3.31 (0.15)	37.77
	Plate	−0.369 (0.020)	11.75 (0.54)	134.11
